# Hydrotreated vegetable oil migrates through soil and degrades faster than fossil diesel and hydrotreated vegetable oil-fossil diesel blend

**DOI:** 10.1007/s11356-024-34760-2

**Published:** 2024-08-23

**Authors:** Katariina Lahti-Leikas, Emilia Niemistö, Harri Talvenmäki, Niina Saartama, Yan Sun, Léon Mercier, Martin Romantschuk

**Affiliations:** Faculty of Biological and Environmental Sciences, University of Helsinki, Niemenkatu 73, 15140 Lahti, Finland

**Keywords:** Hydrotreated vegetable oil, Diesel, Light non-aqueous phase liquid migration in soil, Water accommodated fraction, Biostimulation, Natural attenuation

## Abstract

HVO has been noted as a more sustainable fuel, not only leading to lower total CO_2_ emissions, but also resulting in lower emissions of toxic substances upon fuel burning. The environmental impact of HVO and HVO diesel blends when accidentally spilled into the soil and ground water has, however, received little attention. While HVO and diesel exhibit nearly identical viscosity and density, their behavior in soils differs due to varying water solubility and fuel additives. In laboratory- and pilot-scale soil columns and lysimeters, we compared the migration and biostimulation-enhanced degradation of HVO, HVO-diesel blend (HVO15), and fossil diesel over 120 days. Additionally, we investigated the impact of fuel additives on migration by comparing HVO without additives to HVO15 and diesel in wet and dry soil columns over 21 days. Notably, HVO migrated through soil more rapidly and in greater quantities than diesel. In wet soil, 69% of added HVO, 8.4% of HVO15, and 21% of diesel leached through as light non-aqueous phase liquid (LNAPL). Dry soil showed smaller differences in fuel migration, but HVO did not mobilize when water was added, unlike HVO15 and diesel. Biostimulation reduced HVO leaching by 15% more than HVO15 and 48% more than diesel. Overall, HVO’s behavior in soil differs significantly from fossil diesel, with factors like lower water solubility, reduced mobilization from dry soil, and higher in situ degradability contributing to its reduced environmental risk compared to fossil fuel alternatives in accident scenarios.

**Supplementary Information:**

The online version contains supplementary material available at 10.1007/s11356-024-34760-2.

## Introduction

The high carbon footprint of fossil fuel use, rising oil prices, and efforts to gain independence from imported oil have accelerated the large-scale commercial production of renewable diesel and hydrotreated vegetable oil (HVO) (Hill et al. 2006). HVO has been presented as an alternative that reduces CO_2_ and toxic substances in emissions when used as fuel in internal combustion engines (Di Blasio et al. 2022; McCaffery et al. 2022). A prerequisite for committing to this transition is proper knowledge of the behavior of HVO-based fuels in the engine (Pellegrini et al. 2015), the emissions resulting from combustion (Di Blasio et al. 2022), as well as the behavior of HVO and HVO blends when accidentally released into the environment. In 2024, HVO demand is expected to rise to 18.9 Bln L, respectively (International Energy Agency 2022). The greater volume of production and transportation worldwide increases the chance of accidents during HVO transportation. Approximately 80% of the 10 million tons of hazardous substances transported on Finnish roads annually are fossil oil products, contributing to 150 transportation accidents (Kallio and Mäkelä 2012).

The paraffinic hydrocarbon composition of HVO is similar to that of fossil diesel; however, a few notable differences exist between the two. HVO is a product of hydrotreatment and is compared to fossil diesel, a more homogeneous mixture of paraffinic hydrocarbons, mainly in the range of C10–C20, of which C15–C18 has the highest concentrations (Mikkonen 2008, Aatola et al. 2009, Nylund et al. 2011, McKone et al. 2012, Bezergianni and Dimitriadis 2013, Zeman et al. 2019). Fossil diesel contains paraffinic oil hydrocarbons in a larger range, from C12 to C24, as well as sulfur, and a significantly higher proportion of aromatics (Pál et al. 1998; Aatola et al. 2009; Shamsuddin et al. 2022; Zeman et al. 2019). Compared with fossil diesel, HVO has a higher octanol–water partition coefficient and slightly lower density and volatility (Mikkonen 2008, ITRC 2011, Nylund et al. 2011, Bezergianni and Dimitriadis 2013, Napolitano et al. 2018, Preuß et al. 2021).

Because the key factors governing the subsurface migration of fuels, such as viscosity and density, are almost identical for HVO and fossil diesel, it is expected that their environmental behavior will show little variation (McKone et al. 2012). However, the lower water solubility and volatility of HVO suggest that HVO may have a smaller water-accommodated fraction (WAF) and vapor phase in the soil environment than fossil diesel. Furthermore, additives present in fossil diesel and HVO can lead to variations in how they move through substances (McKone et al. 2012, Bezergianni and Dimitriadis 2013, Preuß et al. 2021). HVO is also likely easier to degrade naturally or via biostimulation-based remediation than fossil diesel and may facilitate the degradation of fossil diesel components in HVO-diesel blends (Hidalgo et al. 2024, Thomas et al. 2017). This seems to be related to the lower presence of aromatics and minimal amounts of other hard-to-break down oil components (> C18) found in fossil diesel (Lacalle et al. 2021; Simpanen et al. 2016; Wilms et al. 2023).

The goal of this research was to examine the variations in environmental behavior between commercially produced HVO, HVO-diesel blend, and fossil diesel after a simulated roadside spill, which commonly involves the discharge of substantial fuel volumes. Sandy soil groundwater areas are at a high risk of contamination from fuel spills (Halmemies et al. 2003), and fuel accidents frequently occur on roads, leading to the exposure of roadside verges to oil hydrocarbons (Kallio and Mäkelä 2012). The current study aims to demonstrate the differences in the behavior of HVO and HVO-diesel blend compared to fossil diesel in accidental spill scenarios in homogeneous sandy soils and heterogeneous roadside verges and the response of the diesels to biostimulation procedures shortly after contamination in the unsaturated zone.

## Materials and methods

In this study, the behaviors of HVO, HVO-diesel bland, and fossil diesel in terms of migration, degradation, and evaporation, as well as the response of the tested fuels to biostimulation amendments in the case of an accidental spill, were investigated. This study consisted of four different experiments (Table 1): laboratory-scale LNAPL migration experiment in wet and dry soil (experiment 1, Table 1), laboratory-scale biostimulation experiment (experiment 2, Table 1), pilot-scale biostimulation experiment in heterogeneous soil (experiment 3, Table 1), and pilot-scale biostimulation experiment in homogeneous soil (experiment 4, Table 1). The migration and biostimulation experiments were separately performed to focus specifically on the migration and distribution of the fuels in the soil and leachate under different soil conditions and the effect of the fuel additives on LNAPL migration. In the migration experiment (experiment 1), the migration of HVO containing no fuel additives, HVO-diesel blend, and diesel in dry soil followed by flushing of the soil, and how the diesels migrate in soil that is wet from the start, followed by additional water flushing for 21 days.

**Table 1:** Experimental descriptions of laboratory- and pilot-scale biostimulation and natural attenuation experiments and laboratory-scale migration experiment^a^

Experiment	Purpose of the experiment	Duration (d) and soil volume	Amount and type of fuel added	Replicates used	Amount of water added	Amount of biostimulation solution added	Monitoring of the leaching and degradation of oil hydrocarbons
Experiment 1: laboratory-scale migration experiment in wet (moisture content 4%) and dry (moisture content 0%) soil	To compare the migration of different fuel types in soils with different moisture content	21 d 0.5 dm^3^	8.3 g each of HVO without additives (HVO0), HVO15, and diesel	4	For wet soil: 40 mL on days 3, 7, 14, and 21. For dry soil: 170 mL on day 7, 40 mL on days 14 and 21		In wet soil migration monitored by analyzing the amount of the leached LNAPL In dry soil migration monitored by measuring the depth of the migrating LNAPL front and analyzing the amount of the leached LNAPL
Experiment 2: laboratory-scale biostimulation experiment	To study the migration behavior and the response of the tested diesels to biostimulation amendments in an accidental spill scenario	120 1.6 dm^3^	20 g each of HVO, HVO15, and diesel	4 replicates each for biostimulation and natural attenuation	50 mL of water added on days 4, 5, 11, 12, 18, 19, 25, 26, 31, 32, 39, 40, 53, 54, 61, 62, 76, 77, 84, 85, 91, 92, 116, and 117	365 mg of urea in 50 mL of water added on biostimulation soil columns and 50 mL of water to natural attenuation soil columns on days 15, 54, and 84	Degradation and migration of the tested diesels monitored by analyzing oil hydrocarbon concentration in soil and leachate water
Experiment 3: pilot-scale biostimulation experiment in heterogeneous soil	To verify the environmental relevance of the migration behavior and the response of the tested diesels to biostimulation in an accidental spill scenario in heterogeneous soil. Natural attenuation was used as control	120 1.7 m^3^	5.0 kg each of HVO, HVO15, and diesel	One replicate for all the treatments	Exposed to natural rain events	320 g of urea added in 10 L of water on biostimulation lysimeters and 10 L of water on natural attenuation soil lysimeters on day 15	Degradation and migration of the tested diesels monitored by analyzing oil hydrocarbon concentration in soil and leachate water and measuring the volume of leached LNAPL
Experiment 4: pilot-scale biotimulation experiment in homogeneous soil	To verify the environmental relevance of the migration behavior and the response of the tested diesels to biostimulation in an accidental spill scenario in homogeneous soil. Natural attenuation was used as control	120 1.7 m^3^	6.5 kg each of HVO, HVO15, and diesel	Two replicates for biostimulation, one replicate for natural attenuation	Exposed to natural rain events	158 g of urea in 10 L of water added on biostimulation lysimeters and 10 L of water on natural attenuation lysimeters on days 15, 54, and 84	Degradation and migration of the tested diesels monitored by analyzing oil hydrocarbon concentration in soil and leachate water and measuring the volume of leached LNAPL

^a^See more details in sections “Laboratory-scale soil columns and pilot-scale lysimeters,” “Soil exposure with fuels,” “Natural attenuation and biostimulation amendments,” “Leachate sampling,” “Soil sampling,” “Chemical analyses,” and “Statistical analysis and mass balance”

A laboratory-scale biostimulation experiment (experiment 2) was performed to test the fate of fuels in the soil, including migration and degradation during bioremediation for 120 days. Pilot-scale experiments (experiments 3 and 4) under realistic field conditions using lysimeters exposed to natural conditions were performed to verify the environmental relevance of the laboratory-scale results. Pilot-scale experiments were conducted using a mixed soil resembling a roadside verge, made up of sandy till and coarse sand layers (experiment 3), and uniform sandy soil (experiment 4) to assess how different soil compositions affect the movement of the tested fuels.

### Laboratory-scale soil columns and pilot-scale lysimeters

For the construction of the soil columns and lysimeters, clean soil provided by the local earth moving companies was used. SGM Consulting Ltd in Kuopio, Finland analyzed the granularity of the soils according to international standard SFS-EN ISO 17892–4:2016 (Table 2). The geotechnical properties were analyzed by Mitta Ltd in Oulu, Finland (Table 2).

**Table 2:** Geotechnical properties of the soils used in the laboratory- and pilot-scale experiments

Experiment	Soil type	Grain size (mm)	*d*50	Portion of silt (%)	Bulk density (g/cm^3^)	Porosity (%)	Hydraulic conductivity (m/s)
Experiments 1 and 2: laboratory-scale migration and biostimulation experiment	Medium sand	0.6–3	0.532	0	1.16	53.3	1.9 × 10^−4^
Experiment 3: pilot-scale biostimulation experiment with heterogeneous soil	Sandy till	0–32	0.897	9.5	*	*	*
	Sandy till	0–90	0.903	10	*	*	*
	Coarse sand	0.02–3.5	0.824	0	*	*	*
Experiment 4: pilot-scale biostimulation experiment with homogeneous soil	Medium sand	0.6–3	0.532	0	1.25	1.25	9.0 × 10^−5^

^*^Could not be analyzed since the weights of the soil layers were not documented during the fill of the lysimeters

**Fig. 1:**
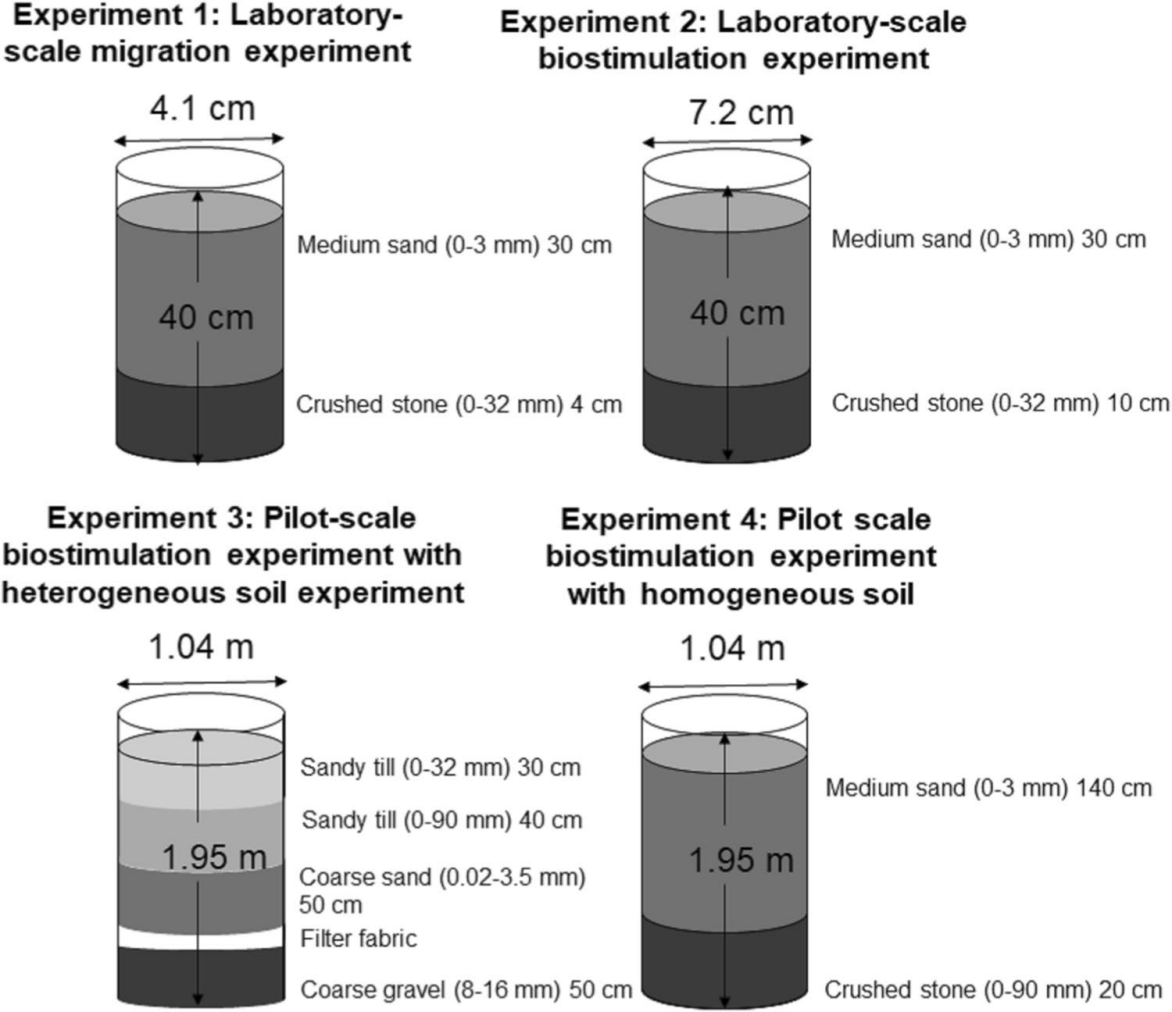
Graphic presentation of the soil composition of the laboratory-scale migration and biostimulation experiments’ soil columns and the lysimeters of the pilot-scale experiment with heterogeneous soil and pilot-scale experiment with homogeneous soil

Laboratory-scale migration and biostimulation experiments (experiments 1 and 2) were carried out in Plexiglas columns (LNAPL migration experiments: height 40 cm and inner Ø 4.1 cm; biostimulation experiments: height 40 cm and inner Ø 7.2 cm), with the bottom ends covered with metal nets (mesh size 3 mm × 3 mm). The columns were filled from the bottom up with crushed stone (particle size 0–32 mm; LNAPL migration experiment: 4 cm; biostimulation experiment: 10 cm), and 30 cm (LNAPL migration experiment: 570 g; biostimulation experiment: 1425 ± 5 g) of sieved medium sand (sieve size 0–3 mm) (Fig. 1). The columns were placed in a fume hood, and glass decanters were placed under each column to collect leachate. To moisten the soil for the laboratory-scale LNAPL migration experiment in wet soil and biostimulation experiments (experiments 1 and 2), 170 and 400 mL of deionized water were added to the columns and allowed to pass through the soil for 2 days before fuel exposure.

The pilot-scale experiments (experiments 3 and 4) were executed at the Soil Research Center SOILIA in Lahti, Finland, using ground-inserted lysimeters (1.7 m^3^, inner Ø 1.04 m, and height 1.95 m) (Simpanen et al. 2016). Six lysimeters with heterogeneous soil constructions simulating roadside verges (experiment 3) were constructed by Kuoppamäki et al. (2019). The lysimeters were filled in June 2014 from the bottom up with 50-cm coarse gravel (8–16 mm), filter fabric, 50-cm coarse sand (0.02–3.5 mm), 40-cm sandy till (0–90 mm), and 30-cm sandy till (0–32 mm) (Fig. 1). A 0.15-m thick layer of growing substrate was added on top of the lysimeters. Kuoppamäki et al. (2019) used the lysimeters for 2 years to study the ability of the organic layer to retain pollutants from artificial stormwater. After the experiment by Kuoppamäki et al. (2019), the lysimeters were left to settle for 1 year. The layers of the growing substrate were removed 3 months before the start of our pilot-scale experiment with heterogeneous soil.

In the pilot-scale experiment with homogeneous soil (experiment 4), nine lysimeters were filled from the bottom up in September 2017 with 20-cm crushed stone (0–90 mm) and 140 cm of the same sieved medium sand (0–3 mm) that was used in the laboratory-scale experiments (Fig. 1). Before fuel exposure, the lysimeters were left to settle for 9 months.

### Soil exposure with fuels

Fuels used in this study were commercial summertime quality fossil diesel, HVO, and an HVO-diesel blend (HVO ≤ 15%, not blended from the same diesel and HVO used in the experiment, referred to as HVO15 in this article) produced by Neste Ltd. (Table 3). HVO in the migration experiments contained only conductivity additives (referred to as HVO0 in this study), whereas the HVO in the biostimulation experiments also contained lubricants. HVO15 and diesel used in all the experiments contained additives. The diesels were transported and stored in metallic canisters and used within 3 months after refinement.

**Table 3:** Physio-chemical properties of the used HVO, HVO15, and diesel. The data was provided by Neste Ltd

Experiment	Fuel	Density (kg/m^3^) at 15 °C	Viscosity (mm^2^/s) at 40 °C	Aromatics (wt%)	Fuel additives
Experiment 1: laboratory-scale LNAPL migration experiment	HVO_0_	779.7	2.883	0.4	Conductivity additive
	HVO15	825.6	2.847	10.7	Lubricity additive, cold flow improver, marketing additive, and conductivity
	Diesel	831.8	2.832	19.5	Lubricity additive, cold flow improver, and conductivity
Experiments 2–4: laboratory- and pilot-scale biostimulation experiments	HVO	779.4	2.872	0.3	Lubricity improver, and conductivity
	HVO15	825.6	2.847	10.7	Lubricity additive, cold flow improver, marketing additive, and conductivity
	Diesel	835.3	2.847	23.2	Lubricity additive, cold flow improver, and conductivity

The volume of the fuels used for the soil contamination was scaled so that the soil layer could retain the spill and no overflow through the soil occurred immediately after the addition of the fuels. The volumes of HVO, HVO15, and diesel compared to the soil mass were higher in the laboratory-scale experiments (experiments 1 and 2) than in the pilot-scale experiments (experiments 3 and 4) to facilitate an even spread of the studied diesels on top of the soil. The volume of the soil in the laboratory-scale migration experiment (experiment 1) was smaller than in the laboratory-scale biostimulation experiment (experiment 2), but the bulk density, porosity, and hydraulic conductivity were equal. In order to reach the same initial fuel concentrations (14 g/kg) 8.3 g of HVO_0_, HVO15, or diesel was added to the smaller, migration test columns (experiment 1) and 20 g of HVO, HVO15 and diesel to the larger, biostimulation and natural attenuation test columns (experiment 2). The topsoil of the pilot-scale lysimeters with heterogeneous soil (experiment 3) was exposed to 5.0 kg, and the second (experiment 4, Table 1) to 6.5 kg of HVO, HVO15, or diesel with a watering can to reach the fuel concentrations 2 and 3 g/kg. A smaller amount of fuel was used to contaminate the soil of the pilot-scale experiment with heterogeneous soil (experiment 3) since the soil types used were coarse although containing also till (Table 2).

### Natural attenuation and biostimulation amendments

In the laboratory-scale biostimulation experiment (experiment 2), four replicates were used for natural attenuation and biostimulation treatment. Due to the limited number of available lysimeters, the pilot-scale experiment with heterogeneous soil (experiment 3) was executed only with one replicate, while in the pilot-scale experiment with homogeneous soil (experiment 4), two replicates were used for the biostimulation treatment and one for the natural attenuation treatment. In the biostimulation treatment the target C:N ratio was set to 100:1 (Kundu et al. 2022; Simpanen et al. 2016). The amount of added urea was calculated according to the soil dry weight, total carbon (TC) concentration, nitrogen (N) concentration, and added diesel oil hydrocarbons. In the laboratory-scale experiment (experiment 2) and in the pilot-scale biostimulation experiments with heterogeneous and homogeneous soils (experiments 3 and 4) 365 mg of urea (Yara Suomi Oy, Finland) in 50 mL of water, 320 g of urea in 10 L of water, and 158 g of urea in 10 L of water were used as a biostimulation amendment. The urea solution was buffered to pH7 with K_2_HPO4 (2.9 mM)/NaH_2_PO_4_ (2.1 mM) buffer. In experiments comparing biostimulation with natural attenuation, the same volume of liquid (nutrients or water, respectively) was added to each column or lysimeter. Biostimulation and rainfall infiltration conditions can decrease the retention of contaminants in low-permeable soils (Thomé et al. 2017). To prevent the mobilization of contaminants in the lysimeters of the pilot-scale experiment with heterogeneous soil (experiment 3), containing low-permeable sandy till the biostimulation amendments were added only once, on day 15. In the laboratory-scale biostimulation experiment (experiment 2) and in the pilot-scale biostimulation experiment with homogeneous soil (experiment 4), the biostimulation amendments were added on days 15, 54, and 84.

### Leachate sampling

In the LNAPL migration experiments (experiment 1) based on preliminary tests, the added water amounts (40/170 mL) was adjusted to generate sufficient leachate quantities for easy separation from LNAPL. In the LNAPL migration experiment in wet soil (experiment 1), the effect of leachate on fuel migration was studied by pouring 40 mL of deionized water on top of the soil 3, 7, 14, and 21 days after the fuel exposure. In the LNAPL migration experiment in dry soil (experiment 1), the LNAPL migration was first monitored by measuring the downward movement of the visible LNAPL front and its distance from the top of the soil column for 7 days. On day 7, 170 mL of deionized water was added to the top of the columns to soak the soil and to generate flushing of the soil by the leachate. Doses of 40 mL deionized water were added on days 14 and 21. The leachate exist the soil within 2 h. The amount of leaching LNAPL mobilized by the leachate was measured gravimetrically by removing the water and weighing the LNAPL remaining in the decanter used for leachate collection.

In the laboratory-scale biostimulation experiment (experiment 2), the effect of the leachate on diesel migration and biostimulation was studied by pouring 50 mL of deionized water on the soil columns twice per day on 2 subsequent days. Starting from the diesel exposure (day 0), water was added on days 4 and 5, 11 and 12, 18 and 19, 25 and 26, 31 and 32, 39 and 40, 53 and 54, 61 and 62, 76 and 77, 84 and 85, 91 and 92, and 116 and 117. The leachate was allowed to pass through the columns into glass decanters (200 mL) for 2 days before storing the water sample at − 20 °C. Because of the small water volume, LNAPL and WAF fractions of the studied diesels could not be separated. Hence, the whole samples (water and LNAPL) were used for the total fossil hydrocarbon analysis.

In the pilot-scale experiments (experiments 3 and 4), the lysimeters were exposed to natural weather conditions, and precipitation was monitored through a weather station placed at the SOILIA Soil Research Center. The leachate passing through the space between the soil and the inner wall of the lysimeter and the leachate passing through the middle of the soil column were collected in two separate 10-L glass flasks. The leachate samples were collected whenever the sampling vessels were full, approximately once a week. The amount of the leachate was monitored by weighing the sampling vessels. The WAF sample was collected first and was taken from the water phase underneath the LNAPL phase by siphoning it into a 90-mL sampling bottle. The LNAPL sample was then separated from the remaining leachate using a separating funnel, and its volume measured with a graduated cylinder. The pH of the leachate samples was measured (Mettler Toledo FiveEasy F20), and the samples were stabilized to pH 2 with HCl and stored at 5 °C.

### Soil sampling

During the LNAPL migration experiments (experiment 1), no soil samples were collected. In the laboratory-scale biostimulation experiment (experiment 2) before the biostimulation amendmentss on days 15 and 85, three soil subsamples were taken from two depths (from top to 15 cm and 15 to 30 cm) with an auger (Ø 12 mm), pooled and stored at − 20 °C. Sampling holes were filled by gently mixing with a glass rod, after which the locations were marked to prevent resampling. On day 120, the entirety of the remaining soil layers was taken as samples.

Because the auger could not pass through the soil in the pilot-scale experiment with heterogeneous soil (experiment 3), the soil samples were taken only at the end of the experiment on day 120. Soil samples were taken by digging the whole soil layer up (from the top towards the bottom 0–30, 30–70, and 70–120 cm), and collecting and pooling multiple small subsamples taken from the same layer. The soil samples and the filter fabrics were stored at − 20 °C.

In the pilot-scale experiment with homogeneous soil (experiment 4), three soil subsamples were taken before each biostimulation amendments (days 15, 54, and 84) from all the lysimeters from three soil layers (from the top towards the bottom 0–45, 45–90, 90–140 cm) with an auger (Ø 50 mm). Subsamples from the same layers were pooled, and stored at − 20 °C. The sampling holes were closed by trampling the walls of the holes, and their locations were marked to prevent resampling. On day 120, the samples were taken as in the pilot-scale experiment with heterogeneous soil from each of the three layers.

### Chemical analyses

Before initiating the experiments, the carbon and nitrogen levels in the soils from the biostimulation trials (experiments 2, 3, and 4) were determined using a C/N/S-analyzer (LECO, CNS-2000) at the Environmental Laboratory and the Faculty of Agriculture and Forestry laboratory of University of Helsinki. The analyses were executed based on the instructions outlined by the manufacturers. In the laboratory- and pilot-scale biostimulation trials (experiments 2, 3, and 4), the pH levels of both water and soil samples were measured with a Mettler Toledo FiveEasy F20 meter. The pH and the dry mass of the soil samples was determined according to ISO 10390:2005—Soil quality — Determination of pH and ISO 11465:1993 Soil quality — Determination of dry matter and water content on a mass basis — Gravimetric method standards.

The total fossil hydrocarbon (TPH) levels in the soil and leachate of laboratory- and pilot-scale biostimulation experiments were analyzed following the international standards Water quality—Determination of hydrocarbon oil index ISO 9377–2:2000 and Soil quality—Determination of content of hydrocarbon in the range C10 to C40 by gas chromatography ISO 16703:2004. Due to the small water sample volume (ca. 100 mL) and soil amount (2.5 g), only 5 mL of extraction solvent (20 mg of *n*-tetracontane and 20 µL of *n*-nonane dissolved into 1000 mL of *n*-hexane) was used. Phase separation for all samples in each experiment was accomplished using a separating funnel. Water and soil extracts of the laboratory-scale biostimulation experiment (experiment 2) and the pilot-scale biostimulation experiment with homogeneous soil (experiment 4) were cleaned with 1.5 g of Florisil and 1.5 g of sodium sulfate. The extracts were stored at − 20 °C until the TPH analysis. Prior to analysis, the soil extracts were diluted 1:10 with the extraction solvent, and the water samples were concentrated to 1:10 using nitrogen gas evaporation.

The TPH concentration of the filter fabrics of the pilot-scale experiment with heterogeneous soil (experiment 3) was analyzed according to ISO 16703:2004 standard at the Environmental Laboratory of University of Helsinki. The filter fabrics were first cut into pieces and soaked in 800 mL of hexane in a shaker for 45 min, after which 400 mL of heptane containing *n*-decane and *n*-tetracontane was added to the vessels. The extraction was washed three times with 400 mL water, dried with Na2SO4, and 10 mL of the dried sample was cleaned with florisil/Na2SO4 cartridges (Sigma-Aldrich PK48 dual layer Florisil/ Na2SO4 PP SPE cartridge).

Quantification of TPH was performed according to ISO 9377–2:2000 and ISO 16703:2004 by using GC-FID (Agilent 6890N) with a Phenomenex’s Zebron ZB-5MS (15 m × 0.25 mm × 0.25 mm) column. The carrier gas was helium; the injector temperature was 350 °C, and the inlet was operated in the splitless mode with an injection volume of 1.0 mL. The oven temperature program was as follows: 50 °C for 2 min, ramp at 20 °C/min to 320 °C, and hold for 10 min. The detector (FID) temperature was set at 340 °C. Calibration was performed using serial dilutions (0.08–10 mg/mL for soil samples and 0.016–10 mg/mL for water samples, *R*^2^ = 0.998) of diesel.

The integration of the samples was done between *n*-nonane (C9) and *n*-tetracontane (C40) in the case of all the soil and water extracts and between *n*-decane (C10) and *n*-tetracontane (C40) in the case of the filter fabric extracts and the TPH concentration of the extracts was calculated following ISO 9377–2:2000 and ISO 16703:2004 standards. In the pilot-scale experiments (experiments 3 and 4) TPH concentration of the water passing through the lysimeter between the soil and the inner wall and through the middle of the lysimeter was summed. Water TPH concentrations and LNAPL volumes were calculated as daily average in each sampling period (days 0–15, days 16–54, days 56–84, and days 85–120).

### Statistical analysis and mass balance

Due to the small number of replicates used in the pilot-scale experiments (experiments 3 and 4), the statistical analysis was executed only for the TPH concentrations of the laboratory-scale experiments’ (experiments 1 and 2) soil and water results. For assessing the differences in the migration in the LNAPL migration experiment (experiment 1) and the differences in the migration and degradation of the different diesels in laboratory-scale biostimulation and natural attenuation soil columns (experiment 2) and between the treatments, the data were analyzed with linear model (lm) statistical test in R (version 3.3.1, package lme4) (R core team 2016). In the statistical analysis of the LNAPL migration experiments (experiment 1), the LNAPL migration depth and leached LNAPL and water volumes were compared. In the laboratory-scale biostimulation experiment (experiment 2) water samples, the effect of the leachate volume and the sampling time on the water TPH concentration was analyzed. In the soil TPH concentration statistical analysis, the effect of soil layer, sampling time and soil pH on soil TPH concentrations was analyzed.

In the laboratory-scale biostimulation experiment and in both of the pilot-scale biostimulation experiments (experiments 2, 3, and 4), a mass balance for TPH was determined by considering each soil column or lysimeter as a control volume. The only source of TPH was the original mass of HVO, HVO15, or diesel present in the contaminated soil. Losses of TPH during the experiments were due to solubility of TPH in percolating water, LNAPL leachate, volatilization, and biodegradation. The TPH remaining in the soil and the TPH leached through the soil were used to calculate the loss of TPH to biodegradation or volatilization.

## Results

### Experiment 1: laboratory-scale LNAPL migration experiments in wet and dry soil

**Fig. 2:**
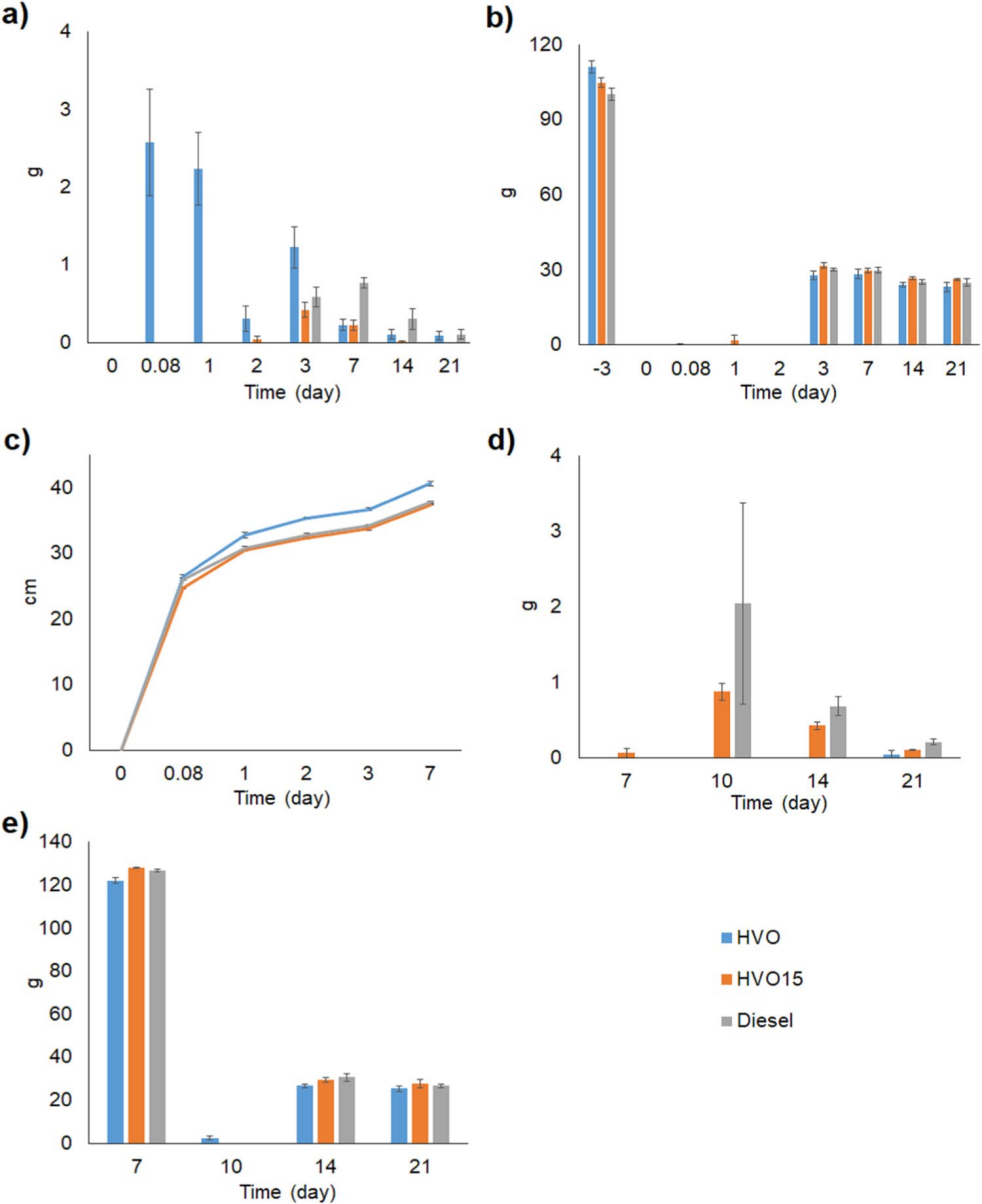
**a** The amount (g ± *SE*) of percolated LNAPL and **b** the amount (g ± *SE*) of percolated water in a migration experiment executed in a wet soil. **c** The migration depth (cm ± *SE*) of LNAPL in a migration experiment executed in a dry soil. **d** and **e** The amount (g ± *SE*) of percolated LNAPL and water since the water rinsing of the soil columns started on day 7 in the migration experiment executed in a dry soil

In the LNAPL migration laboratory-scale experiments (experiment 1), no additives containing HVO_0_ percolated through the wet soil faster and in higher quantities (*p* =  < 0.001, S.1) than the other tested diesels. In wet soil (moisture content 4.3%) ca. 80%, 8.0%, and 21% of the added HVO_0_, HVO15, and diesel were recovered as LNAPL (Fig. 2a). The HVO_0_ LNAPL percolation through the soil was fast, since 30% of the added HVO_0_ leached through the soil within 2 h after HVO_0_ exposure and slightly more than half of the added HVO_0_ within the first 24 h. On the second day, the leaching of HVO_0_ had slowed down, but the addition of water to soak the soil on day 3 re-escalated the HVO_0_ leaching. HVO15 LNAPL was first detected in the leachate water sample on the second day following the diesel exposure, while diesel LNAPL did not leach until after the first water flushing event 3 days after the diesel exposure. After the water soaking events started, an additional 25% of the added HVO_0_ leached out. For HVO15 and diesel LNAPL, the additional leachate amounts were almost 10 and 25% of the added amounts. There was no difference in the amount of percolated water between the soil columns (Fig. 2b, S.1).

In dry soil 2 h after the fuel exposure, there was no difference in the migration depth between the tested diesels (Fig. 2c, p =  < 0.001, S.2). HVO was adsorbed by the soil effectively and only 0.5% of the added HVO was recovered as LNAPL during the whole experiment. Interestingly, flushing the soil with water on days 7, 10, 14, and 21 did not enhance the leaching HVO_0_ LNAPL, whereas 18 and 35% of the added HVO15 and diesel, respectively, was recovered as LNAPL after the flushing events started (Fig. 2d, S.3). The efficiency of water percolation trough the columns exposed to different types of diesel differed so that 5.0% more water from the HVO15 and 4.0% more water from the diesel (*p* = 0.002, S.3) columns had accumulated in the beakers by day 21 after water soaking compared to the HVO results.

### Experiment 2: laboratory-scale biostimulation experiment

**Fig. 3:**
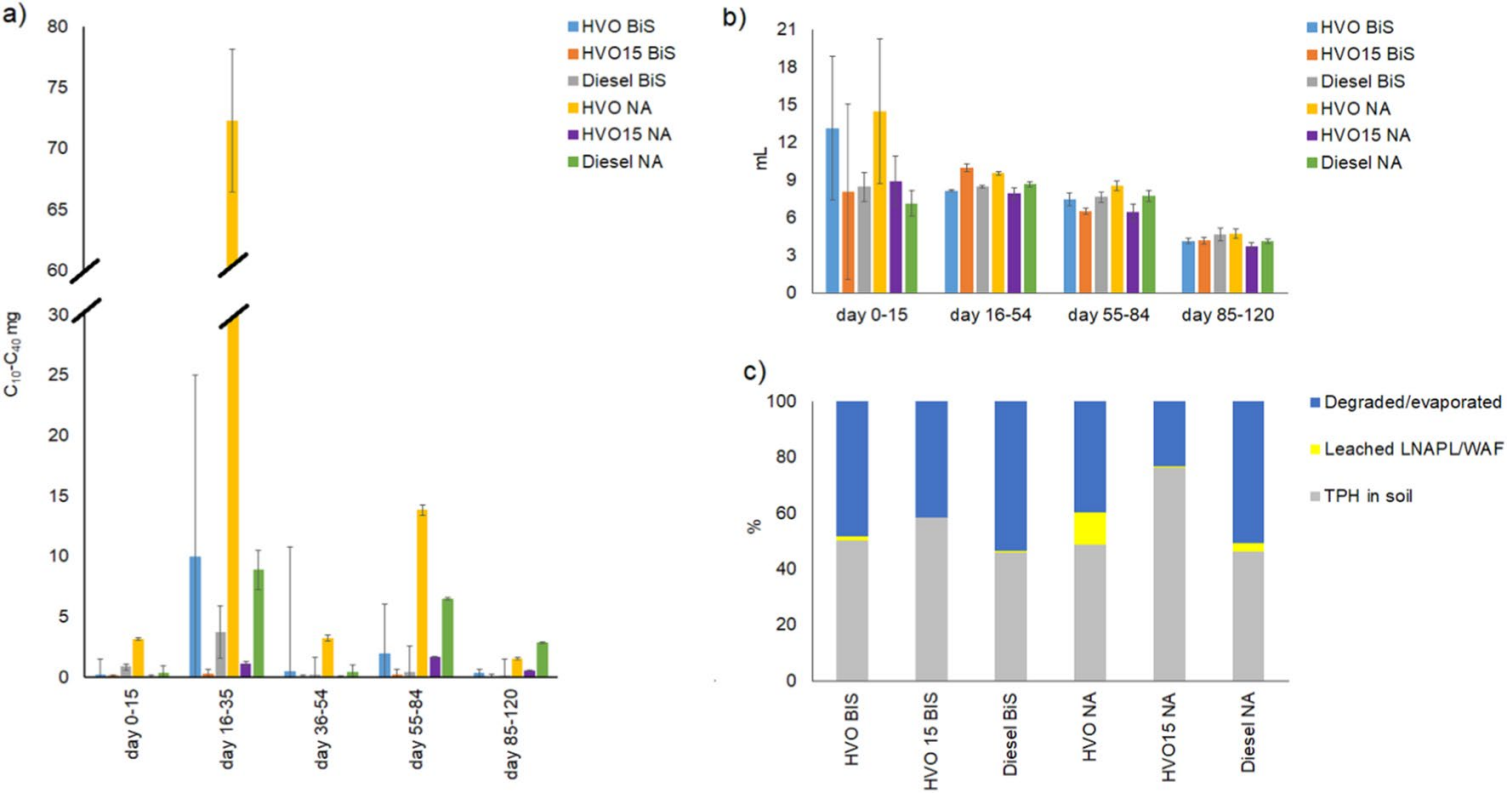
**a** The daily average of leached HVO, HVO15, and diesel amounts in water samples in each sampling period (days 0–15, days 16–54, days 55–84, and days 85–120) (C10–C40 mg, *mean* ± *SE*) in laboratory-scale experiment. **b** The daily average water volumes (mL, *mean* ± *SE*) percolated through the soil in each sampling period (days 0–15, days 16–54, days 55–84, and days 85–12). **c** The percentage of the degraded/evaporated TPH, leached LNAPL/WAF, and TPH in the soil. Biostimulation amendments were added on days 15, 54, and 84

In the laboratory-scale biositmulation experiment (experiment 2) a total of 1.3 and 9.7%, of the added HVO, 0.5 and 0.1% of the added HVO15 and 0.5 and 2.4% of the added diesel leached through the biostimulated and natural attenuated soil, between the first addition of biostimulation amendments or water addition (day 15) and end of the experiment. Compared to HVO15 (*p* =  < 0.001, S.4. Figure 3a) and diesel (*p* =  < 0.001, S.4, Fig. 3a) the leached volumes of HVO were 21- and 7-times higher from the biostimulated soil and 3- and 2-times higher from the natural attenuated soil. Additionally, the leached volume of HVO15 was significantly smaller than that of diesel (*p* =  < 0.001, S.4, Fig. 3a) in both treatments. Biostimulation slowed down the leaching of HVO, HVO15, and diesel for 87, 84, and 80%. The volume of the water percolated through the soil (Fig. 3b) increased the TPH concentration of the leachate (S.4). The pH of the water varied between 5.6 and 7.9.

At the beginning of the laboratory-scale experiment, the soil moisture content varied between 6 and 10%, and the soil nitrogen and organic carbon content was under the detection limit. The biostimulation amendments led to a 48, 41, and 53% reduction of HVO, HVO15, and diesel in soil, whereas the natural attenuation only managed to decrease them by 40, 23, and 51%, respectively (Fig. 3c). However, biostimulation or natural attenuation did not have a significant effect on the TPH concentration of the soil at any depth or at any point during the laboratory-scale experiment nor was there a significant difference between the concentrations of HVO, HVO15, and diesel (S.5).

### Experiment 3: the pilot-scale biostimulation experiment in heterogeneous soil

**Fig. 4:**
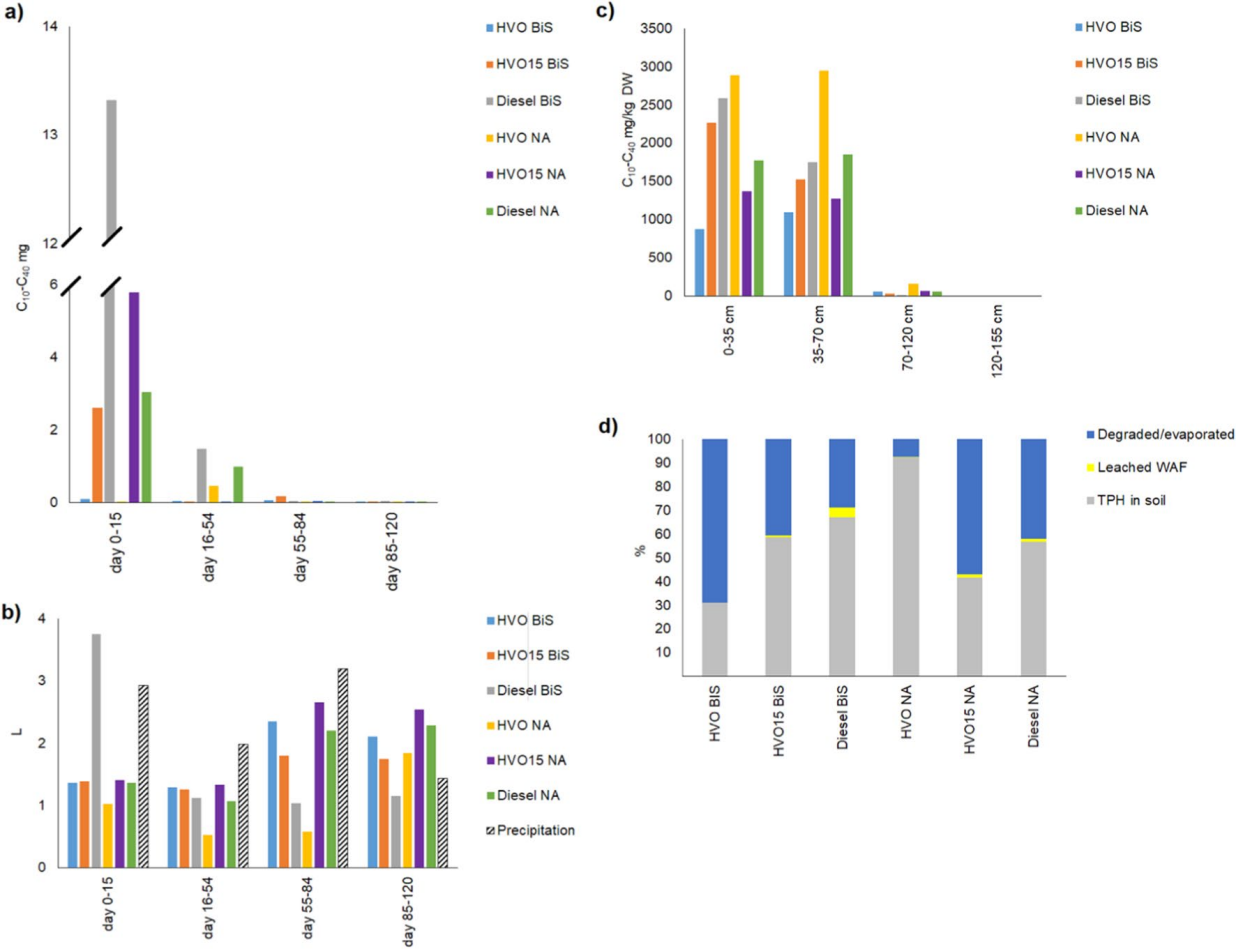
**a** The average daily concentration (C10–C40 mg) of the HVO, HVO15, and diesel WAF in the leachate of the pilot-scale biostimulation experiment with heterogeneous soil in each sampling period (days 0–15, days 16–54, days 55–84, and days 85–120). **b** The average daily leachate (L) and the daily average of precipitation calculated from precipitation records for lysimeter area in each sampling period (days 0–15, days 16–54, days 55–84, and days 85–120). **c** The concentration of TPH (mg/kg DW) on day 120 in soil layers 1 (0–35 cm), 2 (35–70 cm), 3 (70–120), and 4 (120–155 cm) calculated downwards from top of the lysimeter. **d** The percentage of the degraded/evaporated TPH, leached LNAPL, and WAF and TPH in the soil. There were no replicates used in the pilot-scale biostimulation experiment with heterogeneous soil, hence no error bars could be calculated. Biostimulation amendments were added on day 15

In the pilot-scale experiment in heterogeneous soil (experiment 3), no LNAPL phase of HVO, HVO15 or diesel was detected in the leachate. Starting from the addition of biostimulation amendments on day 15, the leachate of the naturally attenuated soil contained nearly 6- and fourfold greater amounts of HVO and HVO15 compared to the leachate of the biostimulated soil. Whereas, the leachate from the biostimulated soil contained 1.5 times more diesel compared to the leachate from the naturally attenuated soil. In the leachate of biostimulated soil, the levels of HVO15 and diesel were 2 and 17 times higher, respectively, compared to HVO, while in the soil exposed to natural attenuation, diesel dissolved to leachate 2-times and HVO15 3-times more than HVO (Fig. 4a). The volume of the leachate (Fig. 4b) correlated with the TPH mass of the leachate. The TPH mass increased with the volume of the leachate until day 50.

In the pilot-scale experiment with heterogeneous soil before fuel exposure, the average of the soil organic carbon concentration was 29.6 g/kg DW (*SE* ± 13.3 g/kg) and total nitrogen concentration 4 mg/kg DW (*SE* ± 0.49 mg/kg). The soil moisture content varied between 2.0 and 6.0% in the soil upper layers and between 6 and 13% in the soil lower layers. Because of the realistic road base simulation, including the pebble and stone containing soil structure and the filter fabric separating layers 3 and 4, the soil could not be sampled using augers, and therefore the soil was sampled only at the end of the experiment upon emptying of the lysimeters.

No TPH was detected in soil layer 4 (120–155 cm) or in the filter fabric between the soil layers 3 (70–120 cm) and 4 in neither of the treatments (Fig. 4c). However, in the soil layers 1 (0–35 cm) and 2 (35–70 cm), the concentration of HVO was nearly three times higher in the natural attenuation than biostimulation lysimeters (Fig. 4c). For HVO15 and diesel, the situation was opposite and TPH concentrations were 1.4 and 1.2 higher in the biostimulated than natural attenuated soil layers 1 and 2. Additionally, in natural attenuated soil, the concentration of HVO was approximately 2 and 1.6 times higher than that of HVO15 and diesel.

In the pilot-scale experiment with heterogeneous soil, the water pH varied between 4.08 and 8.11 and the soil pH was between 6.5 and 7.0 at the end of the experiment. The air temperature varied between + 8.0 and + 19 °C (S. 6).

### Experiment 4: the pilot-scale biostimulation experiment in homogeneous soil

**Fig. 5:**
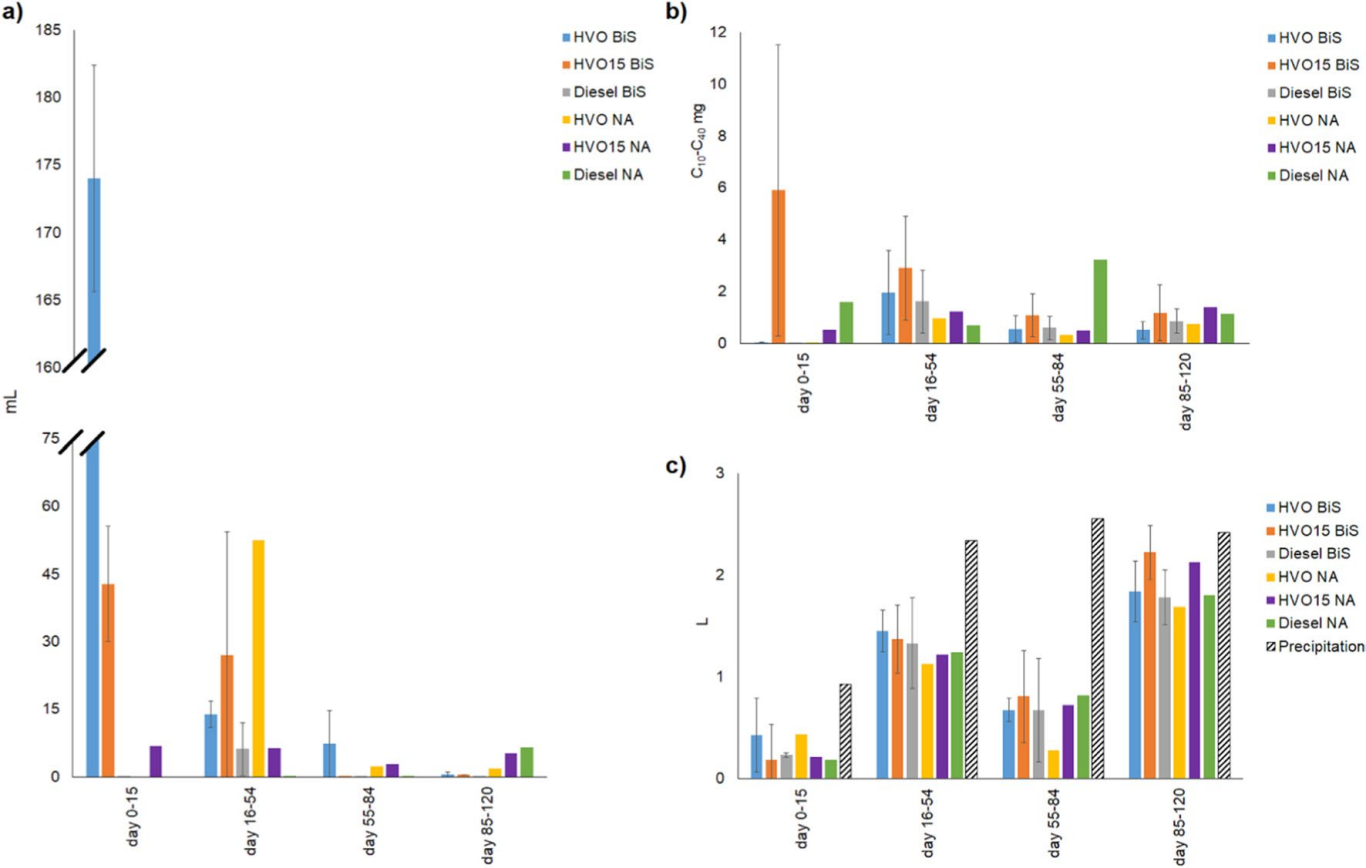
**a** The daily average of HVO, HVO15, and diesel LNAPL volumes (mL, *mean* ± *SE*) in the water samples in the pilot-scale biostimulation experiment with homogeneous soil in each sampling period (days 0–15, days 16–54, days 55–84, and days 85–120). **b** The daily average quantities of the HVO, HVO15, and diesel WAF (C10–C40 mg, *mean* ± *SE*) in the water samples of the pilot-scale biostimulation experiment with homogeneous soil in each sampling period (days 0–15, days 16–54, days 55–84, and days 85–120). **c** The daily average volumes (L, *mean* ± *SE*) of the water percolated through the soil and the daily average of precipitation calculated per lysimeter top soil area (m.^2^) in each sampling period (days 0–15, days 16–54, days 55–84, and days 85–120). Biostimulation amendments were added on days 15, 54, and 84

Before starting the treatments in the pilot scale experiment with homogeneous soil (experiment 4), 40 and 10% of the added HVO and HVO15 were recovered as LNAPL from the lysimeters later to be used for biostimulation, whereas fossil diesel LNAPL was detected in the leachate only after the treatments started. For the lysimeters later to be used for natural attenuation, small amounts of the added diesel LNAPL leached out before the treatments were started, but no HVO or HVO15 LNAPL was detected. After the beginning of the addition of biostimulation amendments on day 15, additional 12 and 35% of the added HVO, 17 and 8.0% of the added HVO15, and ca. 4.0 and 4.0% of the added diesel was recovered as LNAPL from the biostimulated and natural attenuated soil (Fig. 5a).

Following the initial addition of biostimulation amendments on day 15, the concentration of HVO WAF was two times higher in the leachate from biostimulated than natural attenuated soil. For the other diesels, the situation was reversed, with nearly 1.5 to 2.5 times more HVO15 and diesel WAF detected in the leachate of natural attenuated than biostimulated soil. Starting from day 15, the amount of diesel and HVO15 WAF in the leachate from natural attenuated soil was approximately 2 times and 1.5 times higher, respectively, than the amount of HVO WAF In the leachate of biostimulated soil the amount of HVO and HVO15 WAF was 1.5-and 1.1 times higher than the amount of diesel WAF. As expected, the amount of WAF also correlated positively with the volume of percolated water (Fig. 5b, c).

**Fig. 6:**
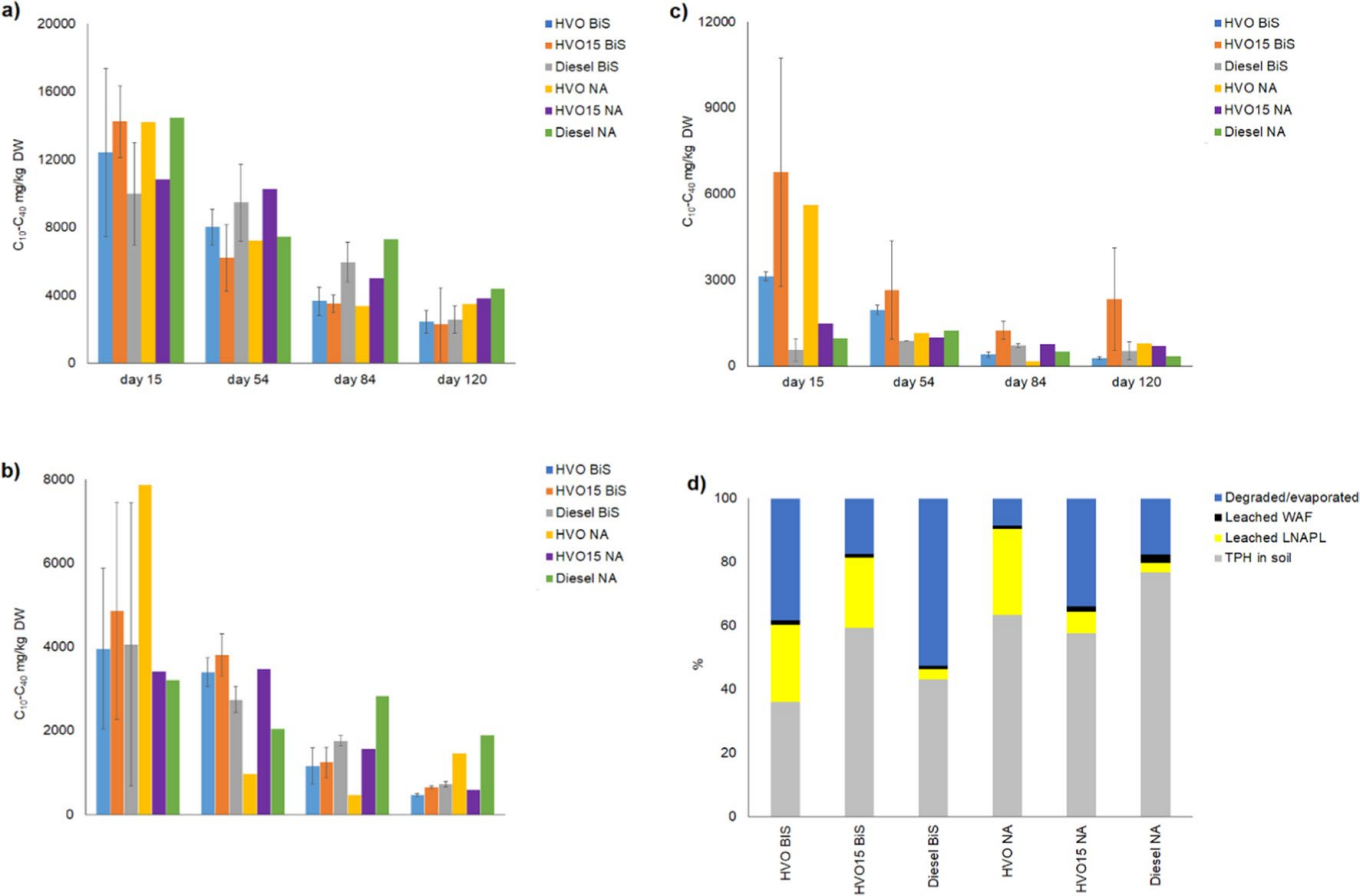
**a** The concentration of TPH (mg/kg DW, *mean* ± *SE*) in the upper (0–30 cm), middle **b** (30–70 cm), and lower layer, **c (**70–120 cm) of the soil in the pilot-scale biostimulation experiment with homogeneous soil. **d** The percentage of the degraded/evaporated TPH, leached LNAPL, and WAF and TPH in the soil. Biostimulation amendments were added on days 15, 54, and 84

At the start of the pilot-scale experiment with homogeneous soil, nitrogen and organic carbon concentrations of the soil were below the detection limit, with moisture content ranging from 4.0 to 6.0% in the soil upper layers and 11 to 13% in the soil lower layers. By the end of the experiment, the concentration of HVO and diesel in the soil subjected to natural attenuation was 1.1 to 1.4 times and 1.1 to 1.2 times higher, respectively, than in the biostimulated soil (Fig. 6a–c). However, for HVO15, the situation was reversed, with the soil TPH concentration being 1.1 to 3 times higher in the biostimulated soil compared to the soil exposed to natural attenuation (Fig. 6a–c). In the biostimulated and natural attenuated soil, the degraded/evaporated percentages of HVO, HVO15, and diesel were approximately 39, 17, 53, and 9%, 34, and 17%, respectively (Fig. 6d). Water pH ranged from 5.3 to 7.8; soil pH varied between 6.4 and 7.1; and air temperatures fluctuated between + 3 °C and + 25 °C. (S. 7).

## Discussion

### LNAPL migration

The migration of light non-aqueous phase liquid (LNAPL) in the subsurface is a function of its physicochemical properties such as density, viscosity, interfacial tension, wettability, and chemical composition, as well as soil properties including porosity, permeability, grain size distribution, moisture content, and heterogeneity (Shen et al. 2023, Waqar 2024). Additionally, the interaction of the released LNAPL with air and water, as well as the LNAPL volume and area of infiltration, duration of release, and subsurface flow conditions affect LNAPL migration (Shen et al. 2023, Waqar 2024). When entering into the soil, gravity causes the LNAPL to migrate downward through the vadose zone as a distinct liquid and some lateral spreading is caused by the capillary forces (Shen et al. 2023, Waqar 2024). As the LNAPL spreads due to its surface tension effects, it leaves residual liquid trapped in the pore spaces, and some of the LNAPL may volatilize and form a vapor extending beyond the LNAPL (Shen et al. 2023, Waqar 2024). The hydraulic conductivity of the soil medium is directly proportional to the density of the migrating LNAPL and inversely proportional to the viscosity of the LNAPL (Malk et al. 2014). Hence, the hydraulic conductivity increases when the fluid viscosity decreases and density increases (Malk et al. 2014). The effect of viscosity can be seen especially between gasoline and diesel: in sand, gasoline migrates 1.5 times deeper and 7–9 times faster than diesel (Malk et al. 2014). In coarser soils LNAPLs move downward through voids between coarse particles when in fine particle soils, owing to capillary forces, the LNAPL flow is rather in the horizontal direction (Alazaiza et al. 2021; Shen et al. 2023). For example, sandy till can retain 1.5–2 times and peat 3.5 times more fuel than gravely sand (Halmemies et al. 2003).

Research on HVO migration in soils is scarce, but in California Renewable Diesel Multimedia Evaluation Final Tier III Report (McKone et al. 2012), the differences in viscosity and density governing the fate and transport of HVO and diesel were assessed to be so similar that no differences in their environmental behavior should be expected. The experiments by Malk et al. (2014) support this notion. Malk et al. (2014) detected no difference in the LNAPL migration between HVO and diesel during a 2-h monitoring in moist and dry soil. McKone et al. (2012), however, raised concerns towards the additives used in HVO. If the additives used in fossil diesel and HVO differ greatly, also differences in the environmental behavior between HVO and diesel could be expected (McKone et al. 2012).

However, in our laboratory-scale and pilot-scale experiments, the difference in LNAPL migration, compared to the HVO15 and diesel, was clear not only for HVO without additives (HVO_0_) but also for the HVO containing additives. Compared to the 2-h migration experiment by Malk et al. (2014), our 21 days’ lasting experiment with regular irrigation (experiment 1) shows that the behavior of HVO_0_ but partly also HVO15 and diesel changes drastically in different soil moisture conditions. In wet soil, ca. one-third of the HVO_0_ had already leached out from the column 2 h after the exposure, while only a small amount of HVO15 LNAPL was detected on the leachate water on day 2, and diesel LNAPL only after the first water flushing event on day 3. The water flushing enhanced the leaching of HVO_0_ and diesel by about the same amount, even though there was much less HVO_0_ left in the column at this point. Notably, the flushing brought out significantly less HVO15 than the other fuel types.

In dry soil, the differences in migration were small (experiment 1). Migration stopped when the hydrocarbons had adsorbed to the soil particles. Interestingly, flushing of the dry soil with water did not result in significant leaching of HVO_0_ unlike for HVO15 and diesel. This retention of HVO_0_ may increase the fuel-water contact surface area and thereby the exposure of HVO_0_ to microbial degradation, which may in part explain why HVO_0_ is more readily biodegradable than the other diesel types. More detailed experiments would be needed to confirm this.

It is known that high molecular weight fractions of fuels alter the wetting characteristics of sandy soils, transforming them from water-wet to oil-wet (Alqam et al. 2021; Al-Ameer et al. 2023). Additionally, additives used in fuels can make the otherwise weakly wetting fuels more wetting (Noruzi et al 2024; Powers et al. 1996). In water-wet systems, LNAPL is entrapped in the largest pore spaces while water is occupying the smallest pore spaces. In an oil-wet system, the situation is opposite, and LNAPL coats the sand grains and occupies the smallest spaces (Alqam et al. 2021; Al-Ameer et al. 2023). The majority of the aromatics in the diesel used in our experiments were monoaromatics (20 wt%), which are relatively water-soluble. Due to the higher concentration of aromatics, the rinsing events in the migration experiment (experiment 1) executed in dry soil enhanced the leaching of HVO15 and diesel but not the leaching of HVO. Being a more hydrophobic product, HVO was not as extractable with water as HVO15 and diesel.

In the pilot-scale experiment with heterogeneous soil, mimicking a real road verge (Kuoppamäki et al. 2019), none of the LNAPL leached out of the lysimeters. It is also notable that by the end of the pilot-scale experiment with heterogeneous soil, most of the TPHs was retained in the lysimeters’ layers 1 and 2, and hardly any TPHs were detected in layer 3, and none in the filter fabric or layer 4. There are several possibilities why LNAPL leached out in high quantities in the pilot-scale experiment with homogeneous soil, whereas none of LNAPL was detected in the leachate water of the pilot-scale experiment with heterogeneous soil. Frist of all, the used amount of fuel for contamination was 1.5 kg lower in the pilot-scale experiment with heterogeneous soil than in the pilot-scale experiment with homogeneous soil. Notably, the soil used in the laboratory-scale experiments and pilot-scale experiment with homogeneous soil contained no silt and the organic carbon concentration was below the detection limit, whereas the soil used in the pilot-scale experiment with heterogeneous soil contained organic carbon ca. 30 g/kg DW and silt (ca. 10%). The organic carbon and the fine silt particles may have retained the oil hydrocarbons so effectively that no free LNAPL leached out from the lysimeters. Indeed, the fabric filter at the third and fourth layer interphase retained fine silt and the silt potentially trapped LNAPL.

Because of large differences in scale, bringing also large differences to wall effects, differences in precipitation and temperature, the possibility for direct comparison of laboratory- to pilot-scale experiments is limited. Nevertheless, in the laboratory-scale migration (experiment 1) and the laboratory- and pilot-scale biostimulation experiments (experiments 2, 3, and 4), the relative amount of leached fuels were similar. HVO leached in significantly higher amounts than the other two fuel types. Perhaps surprisingly, the leaching of HVO15 was significantly smaller than that of diesel. The observation that HVO_0_ LNAPL migrated faster and deeper in wet soil and was not extractable from dry soil by percolating water is important when considering the environmental impact of types of diesel when accidentally spilled. HVO with and without fuel additives migrated faster through soil than the other tested fuel types, showing that fuel additives do not change the HVO LNAPL behavior in this respect.

### Effect of biostimulation on mobile TPH

HVO primarily consists of straight-chain paraffinic hydrocarbons, similar to the structure of natural lipids found in the environment (Knothe 2010; Surger et al. 2023). This structural similarity to natural fatty acids likely enhances HVO’s compatibility with the metabolic pathways of soil microorganisms (DeMello et al. 2007). While the biodegradability of HVO itself remains understudied, it is established that paraffinic hydrocarbons decompose at a rate similar to biodiesel (fatty acid methyl esters, FAME), whereas the more resistant hydrocarbons in fossil diesel, like aromatics, degrade much more slowly (DeMello et al. 2007).

In our experiments the better response of HVO than HVO15 and diesel to biostimulation can be seen in both laboratory- and pilot-scale biostimulation experiments (experiments 2, 3, and 4). In laboratory-scale biostimulation experiment (experiment 2), biostimulation decreased the leaching of HVO by more than 80%. Compared to HVO15 and diesel, 15 and 48% less HVO leached from the biostimulated lysimeters. This is in line with the study by Simpanen et al. (2016), where biostimulated soil leached 7.0–11% less WAF and 19% less LNAPL than soil treated only by natural attenuation.

In the pilot-scale experiment with heterogeneous soil (experiment 3), the leached volume of HVO WAF was several times higher in the lysimeters exposed to natural attenuation than to biostimulation, but for HVO15 and diesel, the effect was vice versa. Nevertheless, in the pilot-scale experiment with homogeneous soil (experiment 4) WAFs of HVO15 and diesel exhibited favorable responses to the biostimulation amendmentss. After the first addition of biostimulation amendments on day 15, almost twice the amount of HVO15 and diesel WAFs leached out from the natural attenuation-treated lysimeters compared to the biostimulated lysimeters. WAF of HVO, on the other hand, did not respond to the biostimulation amendments since roughly equal amounts of HVO WAF leached through the soil in both the biostimulation and natural attenuation treatments. Conclusions regarding total TPH behavior in these lysimeters is, however, difficult since the WAFs only represent a small fraction of the total. Additionally, biostimulation had no positive effect on the leaching of HVO, HVO15, or diesel LNAPL in neither of the pilot-scale experiments (experiments 3 and 4).

However, in the pilot-scale experiment with heterogeneous soil (experiment 3), the better response of HVO to biostimulation can be seen in the higher degraded amount of HVO in soil when compared to natural attenuation treatment. In the pilot-scale experiment with homogeneous soil (experiment 4), the degraded amount of TPH is higher in the soil exposed to biostimulation than in the soil exposed to natural attenuation for all the tested diesels. However, there were large differences between lysimeters in leaching and degradation. The large differences may reflect uneven soil moisture and packing of the soil during the settling of the lysimeters.

The observed better effect of biostimulation in the laboratory-scale experiment (experiment 2) than in pilot-scale experiments (experiments 3 and 4) may have resulted from a temperature closer to optimal (22 °C) in the laboratory (Kebede et al. 2021, Borah and Yadav 2017). In deeper layers of the lysimeters the temperature year around was 5–8 °C. Closer to the surface, the temperature fluctuated according to season. However, in the laboratory the effect of biostimulation was only seen in the mobile fraction of the tested diesels (experiment 2), while in the pilot-scale experiment with heterogeneous soil (experiment 3) biostimulation degreased the leaching of WAF and the fuels’ concentrations in soil. This maybe a consequence of the higher concentration of the fuels used for the soil contamination in the laboratory-scale biostimulation experiment (experiment 2). Due to the higher initial concentration of the tested diesels, microbes had mobile, easier-to-break-down hydrocarbons available for a longer period of time than in the pilot-scale experiments, which is why the hydrocarbon degradation in laboratory-scale soil columns could have been delayed. From an environmental standpoint, the pilot-scale experiments’ results may be more relevant for boreal conditions (Romantschuk et al. 2023). Thus, if the TPHs reach deep soil layers, the biodegradation is slow unless the biostimulation amendments includes warming of deep soil layers, as done by Simpanen et al. (2018).

Biodegradability studies comparing FAME and petrodiesel have also shown that biodiesel can promote the biodegradation of petrodiesel in biodiesel-diesel blends (Gupta et al. 2022; Thomas et al. 2017). Because of the chemical resemblance between HVO and FAME, HVO can also to be expected to enhance the degradation of petrodiesel in HVO-diesel blends. In our experiments HVO15 was not a blend of the HVO and fossil diesel used in this study, which is why we cannot state straightforward that HVO in HVO-diesel blend enhances the degradation of HVO-diesel blend. However, some conclusions can be drawn from the fact that HVO15 responded better to the biostimulation amendments than what fossil diesel did. That is, not only the HVO fraction was degraded but also the diesel components. In our laboratory-scale biostimulation experiment (experiment 2), biostimulation slowed down the leaching of HVO15 29% more than that of diesel. Mass balance comparison showed a ca. 80% increase in the degradation between biostimulation and natural attenuation treatments, with only near negligible increase for diesel. This correlates with the observation that the presence of biodiesel, when the concentration of FAME in the blend is > 20%, enhances the overall biodegradation of the biodiesel-diesel blend because of cometabolic transformation of hydrocarbons (Hidalgo et al. 2024). Additionally, based on our results, it seems that the HVO fraction slows down the migration of the diesel fraction in HVO-diesel blends, since HVO15 migrated slower and in smaller amounts through both the wet and dry soil in our experiments (experiment 1). The mechanism behind this behavior needs to be addressed in the future, especially considering that, both HVO and diesel migrated faster and in higher quantities than the blend, HVO15.

## Conclusions

Our study provides new information related to the environmental fate of diesel fuels produced from renewable resources. Fossil diesel and renewable HVO exist in soil in different phases and the relative abundance and distribution is dependent on the type of diesel and on soil conditions. We conclude that local conditions may significantly influence the relative behavior of types of TPH.

Our study provides new information related to the environmental fate of diesel fuels produced from renewable resources. Fossil diesel and renewable HVO exist in soil in different phases and the relative abundance and distribution is dependent on the type of diesel and on soil conditions. We conclude that local conditions may significantly influence the relative behavior of types of TPH:

- In dry soil conditions HVO is retained in the oil-wet soil, not influenced by water flow. The HVO spill is trapped and removable by excavation and ex situ or on-site treatment.
- In saturated soil, HVO LNAPL moves more rapidly and in larger amounts to the water table compared to diesel or HVO15, resulting in a reduced presence of residual LNAPL, yet due to the decreased solubility of HVO and lower levels of harmful substances, the concentrations of oil hydrocarbons in soil water and groundwater are lower.
- Even though HVO WAF may also require interventions such as in situ biostimulation or pump and treat, our results indicate that it reacts to such treatment faster than the other types of diesels.
- In wet and dry soil conditions, an HVO spill accident requires very different remediation approaches, but in both situations, HVO appears to be treatable or removable with less effort than blends or pure fossil diesel.

Arising essential questions are: (i) how easy and fast is it to characterize an actual accident situation in order to choose the best available technology and (ii) how do blends of different mixing rates complicate the decision-making. These questions need to be addressed in future research and by gathering information from actual cases.

## Supplementary Information

Below is the link to the electronic supplementary material.

Supplementary file1 (XLSX 11 KB)

Supplementary file2 (XLSX 11 KB)

Supplementary file3 (XLSX 11 KB)

Supplementary file4 (XLSX 11 KB)

Supplementary file5 (XLSX 11 KB)

Supplementary file6 (DOCX 20 KB)

Supplementary file7 (DOCX 20 KB)

## Data Availability

The authors confirm that the data supporting the conclusions of this study can be accessed in the paper and its Supplementary Information documents. The corresponding author can provide raw data files in a different format upon a reasonable request.
