# Supplementary material for: Hydrotreated vegetable oil migrates through soil and degrades faster than fossil diesel and hydrotreated vegetable oil-fossil diesel blend
Source: Environ Sci Pollut Res Int. 2024 Aug 23;31(40):53177–92. doi: 10.1007/s11356-024-34760-2 (PMC11379761; doi:10.1007/s11356-024-34760-2)
Supplement: Supplementary file 6 — Supplementary file6 (DOCX 20 KB) [file 11356_2024_34760_MOESM6_ESM.docx]

# **Hydrotreated vegetable oil migrates through soil and degrades faster than petroleum diesel and hydrotreated vegetable oil-petroleum diesel blend**

Environmental Science and Pollution Research

Katariina Lahti-Leikas^a^; Emilia Niemistö^a^; Harri Talvenmäki^a^; Niina Saartama^a^; Yan Sun^a^; Leon Mercier^a^.; Martin Romantschuk^a^

a Faculty of Biological and Environmental Sciences, University of Helsinki, Niemenkatu 73, 15140 Lahti, Finland

Corresponding author: Katariina Lahti-Leikas, Faculty of Biological and Environmental Sciences, University of Helsinki, Niemenkatu 73, 15140 Lahti, Finland ([katariina.lahti@helsinki.fi](mailto:katariina.lahti@helsinki.fi))

**Supplement 6.** The daily average air temperature during pilot-scale biostimulation experiment with heterogeneous soil (Finnish Meteorological Institute, Laune meteorological station 3 km from the experiment area).
